# Combinatorial *BCL2/BCL2L1* expression predicts clinical response to ruxolitinib in myelofibrosis

**DOI:** 10.1186/s40364-025-00865-0

**Published:** 2025-11-24

**Authors:** Giacomo Coltro, Viola Videschi, Francesca Gesullo, Federica Violi, Manjola Balliu, Alessandro M. Vannucchi, Paola Guglielmelli

**Affiliations:** 1 Department of Experimental and Clinical Medicine, University of Florence, Florence, Italy; 2 CRIMM, Center for Research and Innovation of Myeloproliferative Neoplasms, Azienda Ospedaliero-Universitaria Careggi, Florence, Italy; 3 School of Medicine, University of Florence, Florence, Italy

**Keywords:** BCL-2, BCL-xL, BCL-2 family proteins, Combinatorial score, MCL-1, Myelofibrosis, Response prediction, Ruxolitinib

## Abstract

Myelofibrosis is characterized by aberrant JAK/STAT signaling, with approved therapy including the JAK inhibitor ruxolitinib. Preclinical evidence implicates BCL-2 family proteins in MF pathogenesis and therapeutic response. Here, we evaluated baseline and on-treatment expression of *BCL2*, *BCL2L1* (encoding BCL-xL), and *MCL1* in 19 myelofibrosis patients receiving ruxolitinib. Quantitative PCR fold-change (FC) values, relative to healthy donors, revealed reduced baseline *BCL2* (mean FC 0.15) and *MCL1* (0.32) expression, with *BCL2L1* showing a non-significant trend toward upregulation. Baseline *BCL2* and *BCL2L1* expression was significantly higher in patients achieving spleen response (responders; *n* = 7) compared to non-responders (*BCL2*: 0.30 vs 0.07, *p* = 0.0130; *BCL2L1*: 2.73 vs 0.52, *p* = 0.0096). Logistic regression confirmed both as independent predictors of response. We derived a combinatorial score (CS = FC^*BCL-2*^ * FC^*BCL2L1*^), which outperformed individual genes in response prediction, as confirmed in logistic regression analysis (OR, 7.5; *p* = 0.0028). ROC-defined cutoff (0.06) stratified patients by likelihood of response (OR 3.3, *p* = 0.0037). Longitudinal analysis showed no significant overall change in gene expression on ruxolitinib, but responders exhibited BCL2 down-regulation at loss of response (*p* = 0.0419). Overall, these preliminary findings suggest that *BCL2* and* BCL2L1* expression, individually and via a simple CS, predict response to ruxolitinib in myelofibrosis. While limited by small sample size and retrospective design, our data support prospective validation and exploration of BCL-2 pathway modulation as a therapeutic strategy.

**Supplementary Information:**

The online version contains supplementary material available at 10.1186/s40364-025-00865-0.

## To the editor,

Myelofibrosis (MF) is driven by dysregulation of the JAK/STAT pathway, which is critically involved in cell growth, survival, and differentiation [1]. This dependence prompted the development of JAK inhibitors (JAKi), with ruxolitinib (Rux) being the first approved. Emerging evidence implicates the BCL-2 family proteins in MF development and treatment response [2, 3], supported by preliminary evidence of efficacy of combining JAK2 and BCL-xL inhibition [4].

Herein, we investigated baseline and on-treatment expression levels of *BCL2*, *BCL2L1* (encoding BCL-xL) and *MCL1* in 19 MF patients (11 primary MF, 8 secondary MF) treated with Rux. Gene expression was assessed by qPCR on granulocyte cDNA, and expressed as fold-change (FC) using the Ct (2−ΔΔCt) method (additional information in Supplemental Material).

Compared to healthy donors, baseline expression of *BCL2* and *MCL1* was lower in Rux-naïve patients, with mean FCs 0.15 (SEM, 0.05) and 0.32 (SEM, 0.08), respectively. Conversely, *BCL2L1* showed a trend for up-regulation with mean FC 1.33 (SEM, 0.41). No significant correlations were noted between baseline expression and clinical and molecular characteristics, except for higher *BCL2L1* expression in *EZH2*-mutated patients.

**Fig. 1:**
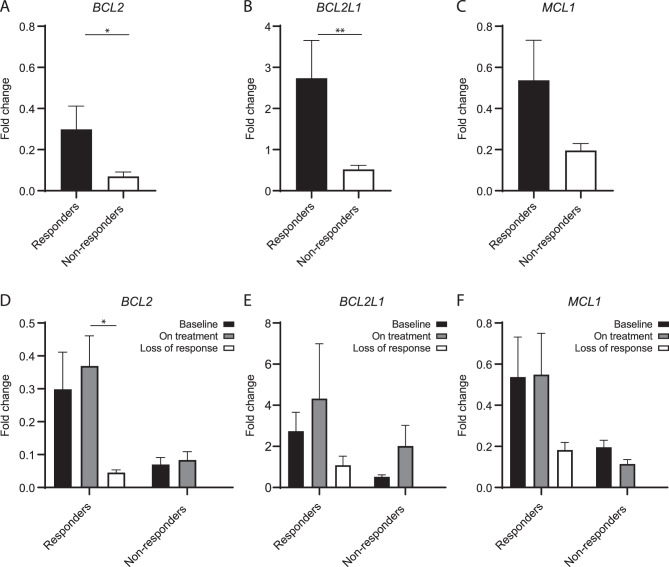
**A-C**. Bar plot of the relative expression of *BCL2* (**A**), *BCL2L1* (**B**), and *MCL1* (**C**) calculated as Fold change at baseline among responder (*n* = 7) and non-responder patients (*n* = 12). **D-F**. Bar plot of the relative expression of *BCL2* (**D**), *BCL2L1* (**E**), and *MCL1* (**F**) calculated as Fold change at baseline-best response-response loss for responder patients and at baseline-on treatment for non-responder patients. Graphs are presented as the mean and SEM of normalized expression values. Statistical significance was determined with ANOVA; *p < 0.05, **p < 0.005, ***p < 0.0005, ****p < 0.0001. *Abbreviations:* SEM, standard error of the mean

Median Rux starting dose was 30 (range, 10–40) mg daily. After a median Rux exposure of 67 (6–121) months, 7 (37%) patients achieved spleen response (“responders”). Upon correlation analysis, *BCL2* and *BCL2L1* were significantly more expressed in responders, with mean FC values 0.30 vs 0.07 (*p* = 0.0130) and 2.73 vs 0.52 (*p* = 0.0096) (Fig. 1A-C). A trend was observed for *MCL-1* (mean FC, 0.54 vs 0.20; *p* = 0.0572). Firth’s logistic regression confirmed that *BCL2* and *BCL2L1* FCs independently predicted response, with respective odds-ratio [OR] of 5.2 (*p* = 0.0337) and 1.4 (*p* = 0.0096).

**Fig. 2:**
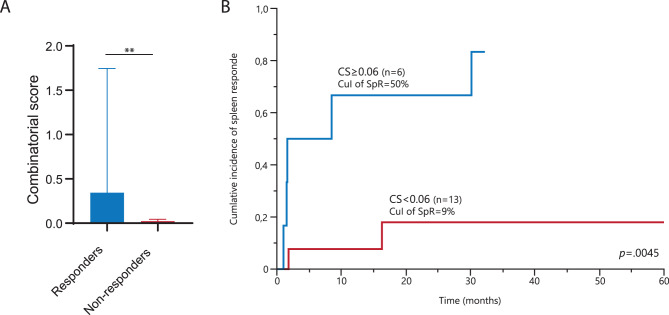
**A**. Bar plot of the combinatorial score (CS) among responder (*n* = 7) and non-responder patients (*n* = 12). **B**. Cumulative incidence of spleen response according to the value of the CS: ≥0.06 (*n* = 6) or < 0.06 (*n* = 13). Graphs are presented as the mean and SEM of normalized expression values. Statistical significance was determined with ANOVA; *p < 0.05, **p < 0.005, ***p < 0.0005, ****p < 0.0001. *Abbreviations:* CS, combinatorial score; CuI, cumulative incidence; SEM, standard error of the mean; SpR, spleen response

Next, we incorporated baseline *BCL2* and *BCL2L1* expression into a combinatorial score (CS) resulting from the product of respective FCs (FC^*BCL-2*^ * FC^*BCL2L1*^). The median CS for responders and non-responders was 0.34 (0.03–4.78) and 0.02 (0–0.32), respectively (*p* = 0.0035) (Fig. 2A). The CS outperformed individual gene expression in predicting Rux response, confirmed in logistic regression analysis (OR, 7.5; *p* = 0.0028). ROC analysis identified 0.06 as the optimal CS cut-off value that was associated with higher probability of Rux response (OR 3.3; *p* = 0.0037), with a cumulative incidence of 24-week response of 50% vs 9% (*p* = 0.0045) (Fig. 2B).

Finally, we prospectively assessed changes in gene expression during Rux treatment (Fig. 1D-F). We found no significant difference between baseline and on-treatment, possibly due to the small sample size. By comparing gene expression of responders at the time of best versus loss of response, we observed a trend for down-regulation for all the three genes at the time of response loss, with a statistical significance for *BCL2* (mean FC 0.08 vs 0.37; *p* = 0.0419). Of 15 patients with longitudinal molecular data available, one non-responder acquired a frameshift *ASXL1* mutation, while one responder developed a non-stop *ASXL1* mutation at response loss.

In summary, we evaluated the expression kinetics and predictive value of *BCL2*, *BCL2L1* and *MCL-1* in a cohort of MF patients treated with Rux. We developed a simple CS that accurately identified responder patients. Recently, Waclawiczek et al. presented a flow cytometry-based “Mediators of Apoptosis Combinatorial Score” (MAC-Score) including the ratio of BCL2, BCL-xL, and MCL1 protein expression in leukemic stem cells [5]; the MAC-Score predicted clinical response to azacitidine/venetoclax with increased event-free survival. Overall, these findings support the potential role of BCL-2 family member expression as predictor of treatment response.

Finally, we observed changes in BCL2 family protein expression during Rux treatment. The small numbers prevented us from obtaining statistical significance, with the exception for *BCL2* expression drop at response loss. Our findings partially contrast with previous data showing higher expression of *BCL2L1* and *MCL1* in refractory/relapsed patients [6]. The counterintuitive kinetics –especially for *BCL2*– may be ascribed to an increased dependence of cell survival on BCL-2 pathway following inhibition of the JAK2/STAT or other pathways. It can also be speculated that the mRNA levels may not accurately reflect changes at the protein level [7]. Finally, we reported the acquisition of two *ASXL1* mutations during Rux treatment. Recently, mutant *ASXL1* was linked to epigenetic upregulation of *BCL2* expression leading to enhanced sensitivity to venetoclax and azacitidine [8]. Interferon-α (IFNα) has also been reported to modulate BCL-2 family protein expression, and its combination with Rux has shown efficacy in MF, thus providing a rationale to explore combined strategies [9, 10].

In addition to their anti-proliferative and pro-apoptotic effects, BCL-2 family inhibitors may also exert relevant anti-inflammatory and immunomodulatory activity. As JAKi suppress cytokine-driven inflammation (a hallmark of MF pathogenesis), combined inhibition could yield synergistic benefit by reducing inflammatory stress and reinforcing apoptotic priming [11].

We acknowledge study limitations, including retrospective design and small study cohort. Notwithstanding, these preliminary data suggest that *BCL2*, *BCL2L1* and *MCL-1* expression may correlate with Rux response. Of note, a *BCL2*/*BCL2L1*-based CS effectively predicted Rux response. Further studies are needed to confirm and expand our data.

## Electronic supplementary material

Below is the link to the electronic supplementary material.

Supplementary Material 1

## Data Availability

The datasets used and/or analysed during the current study are available from the corresponding author on reasonable request.

## Abbreviations

CS: combinatorial score
FC: fold-change
JAKi: JAK inhibitors
MAC-Score: Mediators of Apoptosis Combinatorial Score
MF: myelofibrosis
OR: odds ratio
RUX: ruxolitinib
SEM: standard error of mean
