# Supplementary material for: Combinatorial BCL2/BCL2L1 expression predicts clinical response to ruxolitinib in myelofibrosis
Source: Biomark Res. 2025 Nov 24;13:151. doi: 10.1186/s40364-025-00865-0 (PMC12642052; doi:10.1186/s40364-025-00865-0)
Supplement: Supplementary file 1 — Supplementary Material 1 [file 40364_2025_865_MOESM1_ESM.pdf]

Supplemental Methods

The target genes (i.e., *BCL2*, *BCL2L1*, *MCL1*) were selected based on scientific literature and data from an exploratory analysis performed on a limited number of representative cases using a custom TaqMan® Array Plate including 14 target genes (Supplemental Figure 1). Gene expression of *BCL2*, *BCL2L1* (encoding BCL-xL) and *MCL1* was analyzed by qPCR normalized to endogenous *18S* gene using cDNA obtained from peripheral blood (PB) granulocytes; the relative gene expression (expressed as fold-change; FC) was calculated with reference to healthy controls using the Ct ($2^{-\Delta\Delta Ct}$) method. For the purpose of the study, we attributed ruxolitinib (Rux) response in terms of spleen response, defined as a ≥50% reduction in palpable splenic length lasting ≥6 months.

The study included 19 patients with a diagnosis of primary (n=10) and secondary (n=9) MF who received treatment with Rux. Patient characteristics at Rux start are listed in Supplemental Table 1. Median age was 61 (range, 38-74) years, 10 (53%) were male. Sixteen (84%) patients were *JAK2*-mutated, 2 (11%) *CALR*-mutated, and 1 (5%) *MPL*-mutated.

Six out of 7 responder patients had an available PB sample at the time of best response, while all non-responders had a sample available at some point during Rux therapy. Median time from Rux start and the first sampling time point during treatment was 5.7 (range, 0.8-22.3) months. Among responders, 5 lost their response after a median time of 34 months, and three out of 5 had an available sample for analyses at the time of response loss.

Supplemental Table 1. Main characteristics of the study population (n=19)

Parameter	Value
Male sex; no (%)	10 (53)
Median age at MF diagnosis; years (range)	60.6 (34.8-73.8)
Median age at Rux start; years (range)	61.8 (38.4-74.2)
MF type	
Primary MF; no (%)	11 (58)
Post-polycythemia vera MF; no (%)	3 (16)
Post-essential thrombocytemia MF; no (%)	5 (26)
Median WBC; $x10^9$/L (range)	10.1 (3-57.8)
Median hemoglobin; g/dL (range)	11.1 (7.4-14.4)
Median platelet; $x10^9$/L (range)	210 (97-523)
Constitutional symptoms; no (%)	16 (84)
Spleen >10 cm from LCM; no (%)	15 (79)
Transfusion dependence; no (%)	3 (16)
Median time between MF diagnosis to Rux start; months (range)	27.2 (2.3-109.6)
DIPSS risk category	
Low; no (%)	1 (5)
Intermediate-1; no (%)	5 (26)
Intermediate-2; no (%)	6 (32)
High; no (%)	7 (37)
Mutational status	
JAK2^{V617F} mutated; no (%)	15 (79)
Median *JAK2*^{V617F} allele burden; % (range)	55 (33-98)
CALR mutated; no (%)	3 (16)
MPL mutated; no (%)	1 (5)
Triple negative; no (%)	0 (0)
ASXL1 mutated; n (%)	8 (42)
CBL mutated; n (%)	0 (0)
CSF3R mutated; n (%); evaluable=18	0 (0)
CUX1 mutated; n (%); evaluable=18	0 (0)
DNMT3A mutated; n (%)	0 (0)
EZH2 mutated; n (%)	4 (21)
IDH1/2 mutated; n (%)	0 (0)
KIT mutated; n (%)	0 (0)
KRAS mutated; n (%)	0 (0)
NF-E2 mutated; n (%); evaluable=18	0 (0)
NRAS mutated; n (%)	1 (5)
PTPN1 mutated; n (%); evaluable=18	0 (0)
RUNX1 mutated; n (%); evaluable=18	1 (6)
SETBP1 mutated; n (%); evaluable=18	0 (0)
SF3B1 mutated; n (%)	0 (0)
SH2B3/LNK mutated; n (%)	3 (16)
SRSF2 mutated; n (%)	2 (11)
TET2 mutated; n (%)	2 (11)
TP53 mutated; n (%)	0 (0)
U2AF1 mutated; n (%)	0 (0)
ZRSR2 mutated; n (%); evaluable=18	2 (11)
HMR[‖] mutations[†]; n (%)	10 (53)
≥2 HMR mutations; n (%)	4 (21)
Medain Rux starting dose; mg daily (range)	30 (10-40)
Median Rux exposure time; months (range)	67 (6-121)

Notes: ‖HMR mutations include pathogenic variants in any of the following genes: *ASXL1*, *EZH2*, *IDH1*, *IDH2*, *SRSF2*, and *U2AF1*; ≥2 HMR mutations indicates the presence of 2 or more mutations (2 or more mutations in the same gene are counted as 1)

Abbreviations: DIPSS: dynamic international scoring system; HMR: high molecular risk; LCM: left costal margin; MF: myelofibrosis; Rux: ruxolitinib; WBC: white blood cell.

Supplemental Figure 1

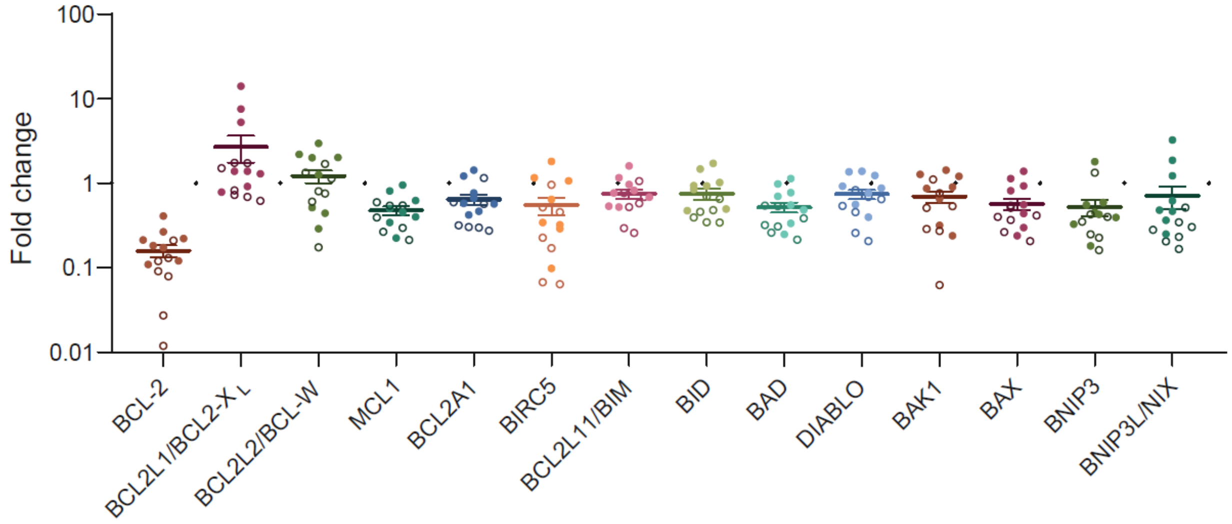

Figure legend

Supplemental Figure 1. Scatter plot of the relative expression of a set of 14 target genes of the BCL-2 family (*BCL2*, *BCL2L1*, *BCL2L2*, *MCL1*, *BCL2A1*, *BIRC5*, *BCL2L11*, *BID*, *BAD*, *DIABLO*, *BAK1*, *BAX*, *BINP3*, *BINP3L*) calculated in 15 ruxolitinib naïve-patients patients with myelofibrosis. Empty dots indicate patients who received treatment with ruxolitinib. Graphs are presented as the mean and SEM of normalized expression values. *Abbreviations:* SEM, standard error of the mean.
